# Mechanisms of Change in the Treatment of Depression: On the Role of Attachment, Reappraisal and Suppression

**DOI:** 10.1002/cpp.70345

**Published:** 2026-09-30

**Authors:** Samy Egli, Theresa Badenbach, Arnold van Emmerik, Angelika Stefan, OPTIMA Study Group, Johannes Kopf‐Beck

**Affiliations:** ^1^ Department Clinical Translation Max‐Planck Institute of Psychiatry Munich Germany; ^2^ Department of Clinical Psychology University of Amsterdam Amsterdam the Netherlands; ^3^ Department of Psychology Ludwig Maximilians University Munich Germany; ^4^ Institute of Population Health, Faculty of Health and Life Sciences University of Liverpool Liverpool UK; ^5^ DZPG (German Center for Mental Health) Berlin Germany

**Keywords:** cognitive behaviour therapy, cognitive reappraisal, depression, expressive suppression, mechanisms of change, schema therapy

## Abstract

Identifying mechanisms of change in psychotherapy is imperative to optimize treatment effectiveness for depression. Research has identified specific mechanisms, primarily in outpatient pathology. However, the transdiagnostic robustness of these mechanisms remains to be quantified in more severely affected populations. This study aims to delineate the role of anxious and avoidant attachment, expressive suppression and cognitive reappraisal as within‐person mechanisms of change in inpatient schema therapy and cognitive behaviour therapy for patients acutely and severely affected by major depressive disorder. A secondary analysis of a randomized clinical trial in an intensive multimodal inpatient and day‐clinic setting with 198 patients was conducted. To differentiate stable between‐person traits from therapeutic within‐person shifts, Bayesian random‐intercept cross‐lagged panel models were employed. The data provided evidence that schema therapy and cognitive behaviour therapy do not differentially affect the mechanisms of change, that these mechanisms do not predict subsequent symptom change and that they do not mediate treatment effects on depressive symptoms. In contrast, a significant reverse association was identified: Symptom reduction was associated with subsequent improvements in cognitive reappraisal and expressive suppression. This temporal precedence suggests that alterations in cognitive reappraisal and expressive suppression are consequences of change in depressive symptoms rather than its primary drivers. These insights could guide researchers and clinicians in future investigations towards precision psychotherapy, directing attention not only to what interventions are performed but also to when they are performed.

## Summary

- Cognitive behavioural therapy (CBT) is the gold standard and schema therapy (ST) a promising emerging treatment for major depressive disorder, but there is limited empirical evidence that explains why they are effective. Obtaining a better understanding of the mechanisms of change by which different treatments operate can help us to improve treatments. Measures of these mechanisms at intake could inform which treatment a patient receives—a pathway to precision psychotherapy. This would mean being able to treat specifically the relevant target for symptom improvement and thereby save time and reduce suffering faster.
- In this study, we investigated four potential mechanisms in an inpatient and day‐clinic psychiatric setting with CBT and ST treatment: improved cognitive reappraisal, less expressive suppression, reduced anxious attachment and reduced avoidant attachment. Patients showed improvements on all four measures from baseline to 6‐month follow‐up. These improvements were comparable for both investigated treatments.
- Our study suggests that improvements in cognitive reappraisal and emotion suppression may occur after an improvement in depression symptoms. This suggests that they are unlikely to be treatment mechanisms that drive symptom change, and instead, a lower depression severity may improve patients' cognitive reappraisal and emotion suppression skills. In practice, this may suggest that skills work should be consolidated once symptoms have started to remit, but this is a hypothesis that needs to be tested in future research.

## 1 Introduction

Depression is a highly prevalent mental disorder that leads to substantial global disease burden, impaired quality of life and high societal costs (Hohls et al. 2021; König et al. 2020; World Health Organization 2023). Recommended treatments for depression are pharmacotherapy and psychotherapy (National Institute for Health and Care Excellence 2022). Cognitive behaviour therapy (CBT) is the current first‐line psychotherapy for depression, yet only about 42% of patients respond to it and 36% achieve remission (Cuijpers et al. 2023). Looking at this population of patients who are affected by a lack of response and remission, that is, looking at patients with chronic or persistent depression, there are two contributing factors attracting attention, that is, with a high prevalence in this population: childhood maltreatment (Nanni et al. 2012; Nelson et al. 2017; Ratzsch et al. 2025) and comorbid personality disorders (Kool et al. 2024). At the same, time childhood maltreatment (e.g., Nanni et al. 2012; Ratzsch et al. 2025) and comorbidity of personality disorders (e.g., Banyard et al. 2021; Newton‐Howes et al. 2014) contribute to an unfavourable treatment outcome in different forms of depression. Schema therapy (ST) is explicitly targeting these two factors (Young et al. 2003) and has recently emerged as a promising alternative treatment (Carter et al. 2013; Kopf‐Beck et al. 2024; Renner et al. 2013). Beyond evaluating such alternative treatments, the efficacy of existing treatments can be further improved by understanding the mechanisms of change (MoC) responsible for their effectiveness (Kazdin 2007): A mechanism is more specific than a mediator in that it is defined as the reason why the change occurred, in other words, how change came about. It is also a mediator, an intervening variable that may account (statistically) for the relationship between the independent and dependent variable. A MoC temporally precedes the outcome, is causally linked with it and therefore is a potential target of the intervention. Consequently, we have identified mechanisms that are particularly relevant in depression and are frequently discussed in the theoretical frameworks of ST and CBT.

Difficulty with emotion regulation is a potential mechanism in the development and persistence of depression and, hence, a key MoC to investigate (e.g., Visted et al. 2018). This review and meta‐analysis on different emotion regulation strategies such as avoidance, rumination, suppression, acceptance, problem solving, reappraisal, awareness, clarity and tolerance found that individuals with current and remitted depression have more difficulties with emotion regulation than never depressed individuals. The authors conclude, that emotion regulation difficulties may continue although depressive symptoms improve, and could contribute to relapse. Two of the just mentioned emotion regulation strategies that received substantial attention in depression research are emotion suppression and cognitive reappraisal (e.g., Rottenberg 2017). Hence, these two specific emotion regulation strategies are going to be the focus of this investigation. Expressive suppression (Steffanowski et al. 2001) refers to efforts to restrain the behavioural or physiological expression of emotions and has been linked to more depressive symptoms. In contrast, cognitive reappraisal, which involves reframing a situation to modify its emotional meaning, has been linked to fewer depressive symptoms (Liu and Thompson 2017). Accordingly, adding emotion regulation training (specifically non‐judgmental perception, acceptance and tolerance, compassionate self‐support, identification of the causes and problem solving as active modification) significantly increases the efficacy of standard CBT in reducing depressive symptoms (Berking et al. 2013). However, to better understand the potentially causal relationship between expressive suppression, cognitive reappraisal and depressive symptoms, it is important to determine if such strategies precede symptom change (Sloan et al. 2017). Radkovsky et al. (2014) indeed showed that the ability to tolerate negative emotions and actively modify undesired emotions was associated with subsequent improvement in depressive symptoms.

Due to their differences in treatment focus, it can be assumed that ST and CBT target different emotion regulation strategies. Specifically, CBT intervenes by cognitive restructuring, which directly targets reappraisal (Clark 2022). Hence, CBT was expected to stronger increase cognitive reappraisal. The literature reviewed below is consistent with this contrast without compelling it. A study of depressed inpatients found that cognitive reappraisal but not expressive suppression improved significantly during CBT, consistent with its cognitive focus (Forkmann et al. 2014).

In contrast, reducing expressive suppression by activating emotions is a key component of ST. Specifically, ST theorizes that emotions arise from patients' maladaptive schemas, and therapists actively support patients to tolerate and express strong emotions without avoiding or suppressing them (Fassbinder et al. 2016; Young et al. 2003). Consistent with this therapeutic focus, difficulties with emotion regulation—as measured by the DERS, that is, the Difficulties in Emotion Regulation Scale (Gratz and Roemer 2004) subscales: (1) non‐acceptance of emotional responses, (2) difficulty in engaging in the goal‐directed behaviour, (3) impulse control difficulties, (4) lack of emotional awareness, (5) limited access to emotion regulation strategies and (6) lack of emotional clarity—mediated the relationship between maladaptive schemas and psychopathology (Yakın et al. 2019). Methodologically, however, this study did rely on a cross sectional non‐clinical community sample, and the mediation model utilized was able to only identify associations between individuals at a single point in time, failing to differentiate stable traits from the within‐person shifts required to identify true mechanisms of change (Kazdin 2007).

As mentioned above, according to schema theory, the therapist actively supports the patient in tolerating strong emotions, targeting the reduction of expressive suppression. The therapist achieves this by providing an opportunity for corrective emotional experiences within a safely attached therapeutic relationship. This is called ‘limited reparenting’, a therapeutic stance in which the therapist acts as a ‘good parent’ within professional boundaries to meet the patient's unmet childhood needs. In ST theory, one of the most central childhood needs is attachment and maladaptive schemas develop based on frustration of such basic psychological needs (Young et al. 2003). Attachment has long been identified as another relevant factor in psychotherapy research (Bowlby 1969, 1980, 1988). Most studies, however, have looked at attachment as a predictor of treatment outcome or baseline characteristic: Meta‐analytic evidence suggests that secure attachment is generally associated with better treatment outcomes (Levy et al. 2018). Regarding depressive symptoms, Dagan et al. (2018) found more depressive symptoms in individuals with insecure‐preoccupied than with secure‐autonomous attachment. Similarly, Conradi et al. (2018) found that insecure attachment predicted an unfavourable course of depression over a 7‐year period. Beyond such associations, one study also showed that secure attachment increases and anxious attachment decreases in adult patients as a result of psychological treatment (Taylor et al. 2015). Taking the central role of attachment in schema therapy (in schema development as well as in treatment) as a starting point and drawing on the definition of a mechanism above, we investigate anxious and avoidant attachment beyond their role as predictors or baseline characteristics in this study as MoC: Does their alteration affect depressive symptoms?

While attachment plays a central role in the psychotherapy in ST, this is not the case in CBT. Here, the most central aspects are cognition and behaviour (e.g., Lorenzo‐Luaces et al. 2016). Fittingly, findings on the effects of CBT on attachment are inconsistent: Whereas McBride et al. (2006) found that patients who scored high on avoidant attachment benefitted more from CBT than from interpersonal therapy regarding depression severity, this has not been replicated by Bernecker et al. (2016).

### 1.1 The Present Study

The present study builds on past research on emotion regulation and attachment, with the specific components cognitive reappraisal, expressive suppression and anxious and avoidant attachment as potential MoCs in psychotherapy for depression. It investigates how these four change during ST compared to CBT and if such change in turn predicts improved depressive symptoms. Clinically, our study could shed light on treatment selection: If ST and CBT act through different mechanisms, measures of these mechanisms at intake could inform which treatment a patient receives—a pathway to precision psychotherapy. This would mean being able to treat specifically the relevant target for symptom improvement and thereby saving time and reducing suffering faster. Based on existing research, we hypothesized that (1) ST leads to a greater reduction in (a) avoidant attachment, (b) anxious attachment and (c) expressive suppression compared to CBT, while CBT leads to a greater increase in (d) cognitive reappraisal compared to ST; that (2) reductions in (a) avoidant attachment, (b) anxious attachment, (c) expressive suppression and increases in (d) cognitive reappraisal predict subsequent symptom improvement; and that (3) the effects of ST and CBT on depressive symptoms are mediated by changes in (a) avoidant attachment, (b) anxious attachment, (c) expressive suppression and (d) cognitive reappraisal. Note that avoidant and anxious attachment and expressive suppression, as maladaptive attachment or coping mechanisms, are targeted to be reduced in order to improve depressive symptoms, whereas cognitive reappraisal is supposed to work the opposite way: As an adaptive coping mechanism, it is aimed to be increased in psychotherapy.

## 2 Method

### 2.1 Design

The present study analysed data from the OPTIMA RCT (OPtimized Treatment Identification at the MAx Planck Institute of Psychiatry, Randomized Clinical Trial), which investigated (among others) ST and CBT for depression in an inpatient and day clinic setting (Kopf‐Beck et al. 2024, 2020). The OPTIMA RCT was registered at clinicaltrials.gov (NCT03287362) and approved by the Institutional Ethic Committee of the Faculty of Medicine of LMU (Ludwig Maximilians University) Munich (Project number 17–395). Its main findings were that high intensity ST for depression as part of a psychiatric care program showed clinical noninferiority compared to CBT, without being superior to IST (individual supportive therapy); regarding short term effects, CBT displayed some advantages over ST, possibly due to its fit with an inpatient and day clinic setting. Overall, ST represents a potentially useful addition to the therapeutic repertoire for depression, but its efficacy, including long‐term efficacy, should be evaluated further. The present study was preregistered on OSF (Open Science Framework, see http://osf.io/r9yfx).

### 2.2 Participants

The study sample consisted of 198 adult participants with a primary diagnosis of depression (52.5% female; mean age *M* = 42.75, SD = 13.37; for a detailed description of the eligibility criteria, see Kopf‐Beck et al. 2020). At baseline, there were *n* = 97 participants in the ST and *n* = 101 in the CBT condition. The assessment dropout rate was 25.3% over the course of 7 weeks. The number of patients at different measurement points is reported in Table 1.

**TABLE 1:** Means and standard deviations of the mechanisms and depressive symptoms.

Variable	Total sample (*n* = 198)			CBT (*n* = 101)			ST (*n* = 97)		
	*n*	*M*	SD	*n*	*M*	SD	*n*	*M*	SD
Baseline (T1)									
DS	198	31.3	8.78	101	30.81	8.44	97	31.80	9.14
ES	194	3.96	1.43	99	3.88	1.40	95	4.04	1.46
CR	194	3.64	1.13	99	3.65	1.21	95	3.63	1.05
AV	192	2.70	0.93	97	2.60	0.94	95	2.80	0.92
AX	192	2.89	0.81	97	2.81	0.83	95	2.97	0.78
Mid‐treatment (T2)									
DS	165	21.4	10.12	90	20.34	9.20	75	22.67	11.15
ES	108	3.65	1.46	59	3.49	1.41	49	3.84	1.52
CR	108	4.01	1.27	59	4.09	1.35	49	3.90	1.16
AV	—	—	—	—	—	—	—	—	—
AX	—	—	—	—	—	—	—	—	—
Post‐treatment (T3)									
DS	148	16.23	10.02	82	14.80	8.99	66	18.00	10.97
ES	139	3.62	1.46	79	3.53	1.47	60	3.73	1.45
CR	139	4.02	1.32	79	4.12	1.47	60	3.89	1.10
AV	138	2.64	0.95	79	2.57	1.00	59	2.73	0.89
AX	138	2.75	0.80	79	2.70	0.82	59	2.81	0.76
6‐month follow‐up (T4)									
DS	108	15.21	12.27	61	13.26	11.41	47	17.74	12.99
ES	86	3.55	1.31	49	3.63	1.33	37	3.45	1.29
CR	86	4.13	1.30	49	4.35	1.31	37	3.84	1.26
AV	86	2.45	0.90	49	2.38	0.94	37	2.53	0.84
AX	86	2.63	0.77	49	2.56	0.79	37	2.74	0.73

Abbreviations: AV = avoidant attachment (RSQ); AX = anxious attachment (RSQ); CR = cognitive reappraisal (ERQ); DS = depression (BDI); ES = expressive suppression (ERQ).

### 2.3 Procedure

Patients were recruited between September 2017 and October 2020 from the inpatient and day clinics at the Max Planck Institute of Psychiatry, Munich. Patients were first diagnosed and screened for eligibility by a physician; those who met the criteria were informed about the study procedure, randomisation and duration by a study psychologist. After providing written informed consent, patients filled in baseline assessments (measurement point T1) and were randomized to a 7‐week treatment arm (measurement point T2 mid‐treatment at week 4 and T3 post‐treatment at week 7), followed by a 6‐month follow‐up assessment (measurement point T4).

### 2.4 Treatments

The interventions of the treatment arms are described according to the template for intervention description and replication (TIDieR) checklist and guide (Hoffmann et al. 2014).

#### 2.4.1 Procedures

ST was delivered in three phases following a manual by Egli et al. (2019): In the first phase, the therapeutic alliance was established, patients were introduced to ST, schemas and schema modes were identified and treatment goals were set. Establishment of the therapeutic alliance was achieved by validation of emotions and needs of the patient, congruent professional self‐disclosure of the therapist, as well as empathic confrontation. Psychoeducation covered how mental disorders develop from a schema therapy perspective, that is by repeated frustration of basic psychological needs (secure attachment, autonomy, freedom to express needs and emotions, spontaneity and play and self‐control), and the rationale of how schema therapy alleviates symptoms and burden, most centrally through corrective emotional experiences by the patient within a safely attached therapeutic relationship. Schemas and modes were identified using questionnaires (Harris 2010; Kriston et al. 2013), as well as highlighting them by the therapist when appearing in the conversation within the therapy session. The second phase focused on changing early maladaptive schemas and dysfunctional schema modes. This was accomplished by employing emotion‐focused techniques like mode dialogues on chairs and imagery rescripting. In imagery rescripting as well as in mode dialogues on chairs, the therapist acts as a ‘good parent’ within the therapeutic stance of limited reparenting. The third phase addressed relapse prevention: A summary of the most important learnings in therapy is created, and the transfer to the everyday life of the patient is developed by identifying potentially difficult situations where schema activations may arise, specifying associated emotions, discriminating biographical experiences from current stimuli and selecting newly learned adaptive behaviours. CBT followed an adaptation of the Hautzinger (2013) manual and included five modules: psychoeducation, behavioural activation, cognitive restructuring, social skills training and relapse prevention. Psychoeducation covered symptoms of depression, models of disorder development according to the stress‐diathesis model and forms of treatment including CBT and psychopharmacotherapy. Specifically, the module cognitive restructuring targets cognitive appraisal and is hence hypothesized to positively influence cognitive reappraisal of emotions; it is based on the ABC technique (Activating Event, Belief, Consequence), elaborated towards a five‐column technique including probabilities and pro and contra arguments. In behavioural activation, value‐based positive activities were chosen and scheduled in the daily routine. In social skills training, social situations related to asserting rights, managing relationships, asking for sympathy and mastering the disorder were exercised in role plays with structured feedback by patients and therapists. Relapse prevention comprised a summary of the most important learnings, their transfer to the patients' everyday routine and the recognition of early warning signs.

#### 2.4.2 Therapists

The treatments were delivered by clinical psychologists or psychiatrists exclusively allocated to either ST or CBT (*n* = 29 and *n* = 28) for a minimum of a year, trained in the particular approach by internationally renowned therapists and supervised monthly by experts in groups of four, separated by condition.

#### 2.4.3 Dose and Format

All patients received two group sessions (100 min each) per week in groups of maximally eight participants provided by two therapists and two individual sessions (50 min each) per week over 7 weeks, amounting to a planned maximum of 28 sessions. Patients received on average 20.62 (SD = 8.57) sessions in ST and 22.76 (SD = 6.14) in CBT, and the dose did not interact with treatment arm in predicting symptom change (Kopf‐Beck et al. 2024).

#### 2.4.4 Tailoring and Modifications

Both manuals were semi‐structured, individualization being limited to the schemas and modes identified for the particular patient in ST and to the transfer of group content into the individual sessions. No planned adaptations were made, except that 24.66% of the trial sample was treated under pandemic hygiene conditions.

#### 2.4.5 Adherence

All sessions were videotaped, and a random subsample of 226 videos (4.1%) was rated by two independent blinded external raters on a 13‐item 6‐point scale (overall ICC = 0.88). Adherence was high to very high and differed highly significant between arms on treatment‐specific techniques, indicating clear treatment differentiation (Kopf‐Beck et al. 2024).

### 2.5 Measures

#### 2.5.1 Depression Severity

The primary outcome was the severity of depressive symptoms, assessed with the German version of Beck's Depression Inventory II (BDI‐II; Hautzinger et al. 2009), which is a widely used self‐report questionnaire of depressive symptoms, demonstrating high internal consistency and good discriminant validity (Kühner et al. 2007). In the present study, internal consistency was high at all four measurement occasions (Cronbach's alpha 0.84–0.93).

#### 2.5.2 Attachment

Attachment was assessed with the German version of the Relationship Styles Questionnaire (RSQ; Steffanowski et al. 2001). This study focused on the subscales ‘fear of closeness’ (7 items) and ‘fear of separation’ (10 items) as these correspond to the avoidant attachment and anxious attachment domains that are the focus of the present study. The internal consistency of the subscales was high at all four measurement occasions (Cronbach's alpha 0.82–0.85).

#### 2.5.3 Cognitive Reappraisal and Expressive Suppression

Cognitive reappraisal and expressive suppression were assessed with the German version of the Emotion Regulation Questionnaire (Abler and Kessler 2009). The ERQ consists of 10 items measuring cognitive reappraisal (6 items) and expressive suppression (4 items). Internal consistency in the present study was consistently high (Cronbach's alpha 0.74–0.92).

### 2.6 Statistical Analyses

Following recent recommendations and publications (e.g., Baljé et al. 2025) for studying MoC in psychotherapy, we used a random‐intercept cross‐lagged panel model (RI‐CLPM) for statistical analysis (Falkenström et al. 2022; Hamaker et al. 2015; Mulder and Hamaker 2021). The RI‐CLPM models the co‐development of variables across longitudinal measurement occasions. It estimates both cross‐lagged and autoregressive effects (Hamaker et al. 2015). Additionally, a random intercept parameter allows disentangling stable, trait‐like differences between persons from within‐person changes (Hamaker et al. 2015; Zyphur et al. 2020), which makes the approach particularly suited to investigate MoC.

We estimated separate RI‐CLPMs for each MoC (anxious/avoidant attachment, expressive suppression, cognitive reappraisal) and depressive symptoms across four measurement points (i.e., T1 = baseline, T2 = mid‐treatment at week 4, T3 = post‐treatment at week 7, T4 = follow‐up after 6 months). The treatment condition was dummy‐coded and added as a two‐level predictor, with paths from treatment to the MoC as well to depressive symptoms for all measurement points after baseline. Note that since the RSQ was not assessed at T2, the RI‐CLPMs for anxious and avoidant attachment were estimated with three time‐points. The model with four measurement points is depicted in Figure 1. Note though that the intervals between measurements are unequal (4 weeks, 3 weeks, 6 months), so cross‐lagged estimates refer to these particular intervals rather than to temporal equi‐distance.

**FIGURE 1:**
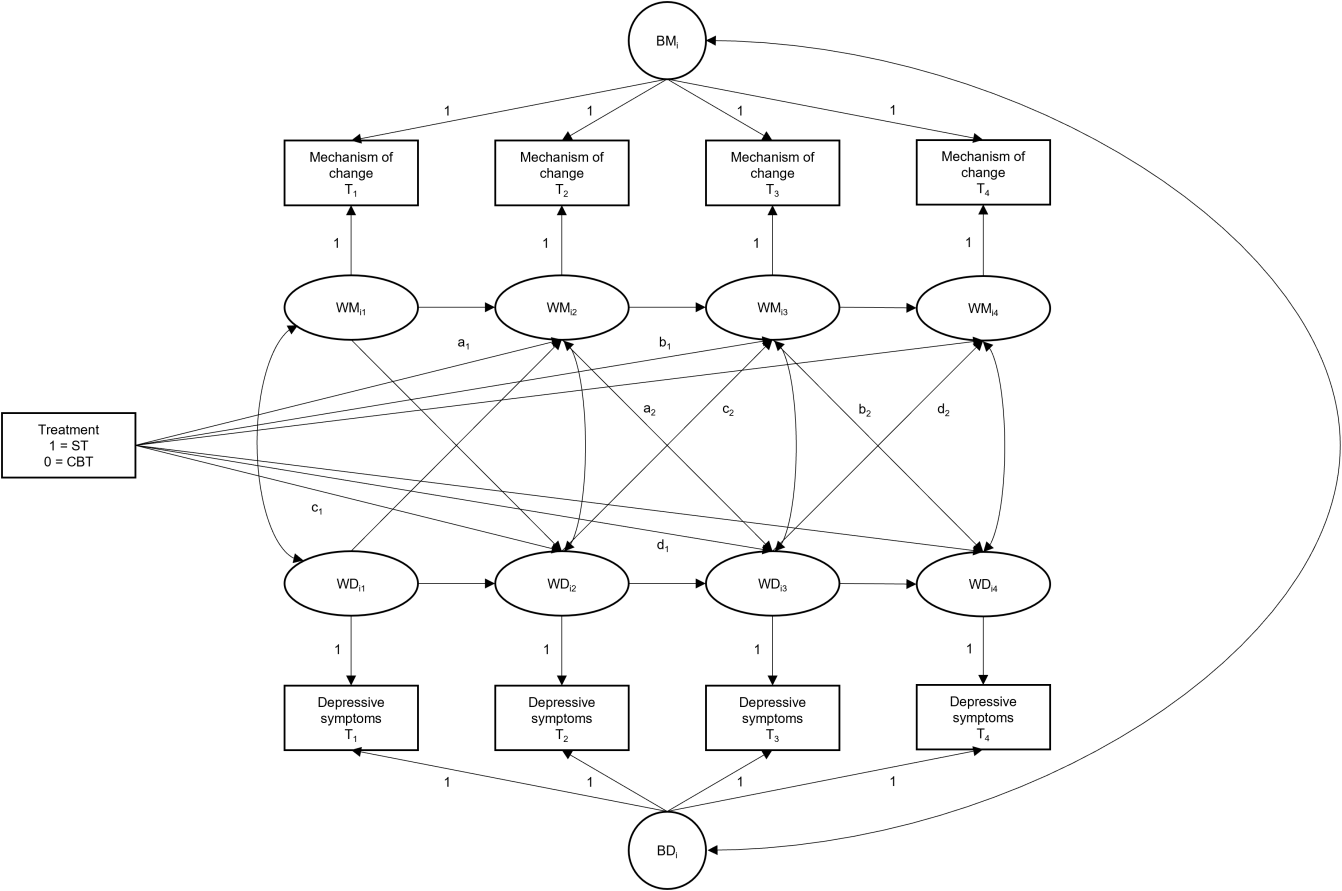
Conceptual representation of the RI‐CLPM between the mechanism of change and depressive symptoms with treatment added as a predictor. *Note:* The latent variables ‘WM’ and ‘WD’ are the within‐person centred variables. ‘BM’ and ‘BD’ represent the random intercepts, distinguishing between‐person differences. M and D refer to the MoC and depressive symptoms respectively. The models were estimated separately for each of the four proposed mechanisms of change (i.e., avoidant attachment, anxious attachment, expressive suppression, cognitive reappraisal). The indirect effects were computed by multiplying the marked paths (e.g., a = a_1_ * a_2_). The indirect effects a and b represent the hypothesized mediation model. The effects c and d represent the reverse mediation model. For avoidant attachment and anxious attachment, only two indirect effects were computed as only three measurement waves were available. Note that the paths for the variances are not depicted for better visibility.

#### 2.6.1 Statistical Power

The original trial was powered for effectiveness testing (Kopf‐Beck et al. 2020). For the current secondary analysis, a post hoc power analysis was conducted in R with the SemTools package (Jorgensen et al. 2022), which uses the Satorra‐Saris method for power calculations (Jak et al. 2021; Satorra and Saris 1985). For α = 0.05, our sample size of *N* = 198 yielded an acceptable power of 0.8 for detecting autoregressive effects of 0.4 and cross‐lagged effects of 0.2, which are comparable to effects found in prior studies (e.g., Lange et al. 2021; Quigley et al. 2018; Radkovsky et al. 2014). The code with the full specification of the power analysis can be found on https://osf.io/5c3pq/overview.

#### 2.6.2 Main Analyses

The pre‐registered maximum likelihood estimation to fit the models revealed Heywood cases (Farooq 2022; Martin and McDonald 1975). The most likely reason was a combination of missing data and low sample size in comparison to model complexity (see our trouble shooting report in Appendix S2). To resolve the issue of negative variance estimates, we deviated from the preregistration and used the Bayesian statistical framework for model fitting (Martin and McDonald 1975). Robustness checks using cross‐lagged panel models without random intercepts can be found in Appendix S1.

Bayesian models were fitted in blavaan (R) using weakly informative priors. We reported posterior means, SDs and 95% credible intervals. Model selection (M1–M3) relied on LOO cross‐validation, with Bayes factors for parameter testing. Notably, Bayes factors quantify the evidence that data provide for the null or alternative hypothesis, respectively, with Bayes factors over 10 conventionally indicating strong evidence (van Doorn et al. 2021). Outcomes were normalized to a 0–1 scale. For Hypothesis 1 (CBT and ST have differential effects on anxious and avoidant attachment and on expressive suppression and cognitive reappraisal), we evaluated the Bayes factors for the paths from treatment to the mechanisms at each time point. To test Hypothesis 2 (changes in the MoC predict changes in depressive symptoms), we evaluated the Bayes factors of the cross‐lagged paths from the mechanism to depressive symptoms at the subsequent time point as well as the opposite paths from depressive symptoms to the mechanism. To answer Hypothesis 3 (the MoC mediate treatment effects on depressive symptoms), the indirect ‘mediation’ paths were computed by multiplying the effect from treatment to the MoC at T2 (path a_1_) with the effect from the MoC at T2 to depressive symptoms at T3 (path a_2_) and from treatment to the MoC at T3 (path b_1_) with the effect from the MoC at T3 to depressive symptoms at T4 (path b_2_) (see Figure 1).

## 3 Results

### 3.1 Descriptive Statistics

Means and standard deviations for depressive symptoms and the MoC at each measurement point are reported in Table 1. The mean scores for depressive symptoms at baseline indicated severe levels of depression. Depressive symptoms decreased over the entire study period: At the 6‐month follow‐up, mean depressive symptoms indicated mild depression. The mechanisms of change also changed in the hypothesized directions: Expressive suppression, avoidant attachment and anxious attachment decreased from baseline to follow‐up. Cognitive reappraisal increased from baseline.

### 3.2 Model Selection

Table 2 shows the results of the model comparisons using the LOO criterion. M3, the least complex model, consistently had the highest predictive accuracy. However, it should be noted that in all analyses the differences are smaller than one standard error. Below, we report estimation and hypothesis testing results in M3. Besides its predictive accuracy, the model provides the most straightforward correspondence between model parameters and hypotheses, because regression weights indicating relationships between postulated MoC and depressive symptoms can be interpreted independently of treatment; in other words, the path between MoC and depressive symptoms is not influenced by treatment.

**TABLE 2:** Results of the model comparisons using the LOO criterion.

Model		LOOIC estimate	SE
Expressive suppression	Unconstrained model	491.6	27.4
	Constrained model	496.0	27.6
	Treatment as predictor model	510.9	29.9
Cognitive reappraisal	Unconstrained model	504.0	27.7
	Constrained model	511.7	28.0
	Treatment as predictor model	520.8	30.6
Avoidant attachment	Unconstrained model	354.4	21.1
	Constrained model	358.8	20.9
	Treatment as predictor model	367.2	22.4
Anxious attachment	Unconstrained model	380.1	29.2
	Constrained model	383.0	30.4
	Treatment as predictor model	396.8	34.4

### 3.3 Hypothesis 1: Treatment Effects

The first hypothesis postulated that ST leads to greater reductions in (a) avoidant attachment, (b) anxious attachment and (c) expressive suppression compared to CBT, while CBT predicts greater increases in (d) cognitive reappraisal compared to ST. Evaluation of the Bayes factors for the paths from treatment to the mechanisms revealed moderate to strong evidence for the null hypothesis on all paths, BF_01_ = 5.41 to BF_01_ = 125. The path estimates are reported in Table 3. Thus, our data provide evidence against Hypothesis 1, indicating that ST and CBT do not differ in their effects on avoidant and anxious attachment, expressive suppression and cognitive reappraisal.

**TABLE 3:** Path estimates of the RI‐CLPMs.

Model	Path	Estimate	BF10	BF interpretation
Expressive suppression				
Autoregressive paths	ES T1 → ES T2	0.470	1.458	Inconclusive evidence
	ES T2 → ES T3	0.619	322.032	Strong evidence for H1
	ES T3 → ES T4	0.078	0.052	Strong evidence for H0
	DS T1 → DS T2	0.358	0.456	Inconclusive evidence
	DS T2 → DS T3	0.662	35,555.177	Strong evidence for H1
	DS T3 → DS T4	0.720	1335.697	Strong evidence for H1
Cross‐lagged paths	ES T1 → DS T2	0.197	0.201	Moderate evidence for H0
	ES T2 → DS T3	0.017	0.026	Strong evidence for H0
	ES T3 → DS T4	0.155	0.111	Moderate evidence for H0
	DS T1 → ES T2	−0.425	0.363	Inconclusive evidence
	DS T2 → ES T3	0.332	0.649	Inconclusive evidence
	DS T3 → ES T4	0.520	6.702	Moderate evidence for H1
Treatment effects	Treatment → ES T2	0.052	0.045	Strong evidence for H0
	Treatment → ES T3	−0.000	0.010	Strong evidence for H0
	Treatment → ES T4	−0.047	0.028	Strong evidence for H0
	Treatment → DS T2	0.034	0.026	Strong evidence for H0
	Treatment → DS T3	0.031	0.023	Strong evidence for H0
	Treatment → DS T4	0.022	0.013	Strong evidence for H0
Cognitive reappraisal				
Autoregressive paths	CR T1 → CR T2	0.074	0.089	Strong evidence for H0
	CR T2 → CR T3	0.278	0.303	Moderate evidence for H0
	CR T3 → CR T4	0.450	1.589	Inconclusive evidence
	DS T1 → DS T2	0.306	0.312	Moderate evidence for H0
	DS T2 → DS T3	0.645	1407.814	Strong evidence for H1
	DS T3 → DS T4	0.716	63.323	Strong evidence for H1
Cross‐lagged paths	CR T1 → DS T2	−0.141	0.086	Strong evidence for H0
	CR T2 → DS T3	−0.036	0.031	Strong evidence for H0
	CR T3 → DS T4	−0.089	0.056	Strong evidence for H0
	DS T1 → CR T2	0.082	0.097	Strong evidence for H0
	DS T2 → CR T3	−0.552	9.769	Moderate evidence for H1
	DS T3 → CR T4	−0.105	0.078	Strong evidence for H0
Treatment effects	Treatment → CR T2	−0.012	0.013	Strong evidence for H0
	Treatment → CR T3	−0.015	0.011	Strong evidence for H0
	Treatment → CR T4	−0.089	0.185	Moderate evidence for H0
	Treatment → DS T2	0.032	0.021	Strong evidence for H0
	Treatment → DS T3	0.034	0.027	Strong evidence for H0
	Treatment → DS T4	0.019	0.013	Strong evidence for H0
Avoidant attachment				
Autoregressive paths	AV T1 → AV T3	0.532	1.758	Inconclusive evidence
	AV T3 → AV T4	0.363	0.702	Inconclusive evidence
	DS T1 → DS T3	0.140	0.094	Strong evidence for H0
	DS T3 → DS T4	0.708	444.585	Strong evidence for H1
Cross‐lagged paths	AV T1 → DS T3	−0.028	0.061	Strong evidence for H0
	AV T3 → DS T4	0.143	0.080	Strong evidence for H0
	DS T1 → AV T3	−0.148	0.082	Strong evidence for H0
	DS T3 → AV T4	0.282	0.215	Moderate evidence for H0
Treatment effects	Treatment → AV T3	−0.007	0.009	Strong evidence for H0
	Treatment → AV T4	−0.035	0.019	Strong evidence for H0
	Treatment → DS T3	0.045	0.047	Strong evidence for H0
	Treatment → DS T4	0.019	0.013	Strong evidence for H0
Anxious attachment				
Autoregressive paths	AX T1 → AX T3	0.382	0.277	Moderate evidence for H0
	AX T3 → AX T4	0.020	0.066	Strong evidence for H0
	DS T1 → DS T3	−0.001	0.077	Strong evidence for H0
	DS T3 → DS T4	0.767	243.687	Strong evidence for H1
Cross‐lagged paths	AX T1 → DS T3	0.309	0.184	Moderate evidence for H0
	AX T3 → DS T4	−0.074	0.064	Strong evidence for H0
	DS T1 → AX T3	0.122	0.082	Strong evidence for H0
	DS T3 → AX T4	0.385	0.396	Inconclusive evidence
Treatment effects	Treatment → AX T3	−0.005	0.008	Strong evidence for H0
	Treatment → AX T4	−0.007	0.011	Strong evidence for H0
	Treatment → DS T3	0.050	0.067	Strong evidence for H0
	Treatment → DS T4	0.017	0.013	Strong evidence for H0

### 3.4 Hypothesis 2: Cross‐Lagged Effects

Hypothesis 2 stated that improvements in (a) avoidant attachment, (b) anxious attachment, (c) expressive suppression and (d) cognitive reappraisal predict subsequent symptom improvement, suggesting a causal role of these improvements in therapeutic change. For all paths from the MoC to depressive symptoms Bayes factors indicated moderate to strong evidence for the null hypothesis, BF_01_ = 4.98 to BF_01_ = 38.46. Thus, Hypothesis 2 was not supported. However, considering the reverse associations, moderate evidence in favour of the alternative hypothesis was found from depressive symptoms at post‐treatment to expressive suppression at the 6‐month follow‐up, BF_10_ = 6.70, β = 0.52, as well as from depressive symptoms at mid‐treatment to cognitive reappraisal at post‐treatment, BF_10_ = 9.77, β = −0.55 (see Table 3). Hence, the results indicate that changes in the proposed mechanisms do not predict changes in depressive symptoms; in contrast, increases in cognitive reappraisal and reductions in expressive suppression followed reductions in depressive symptoms.

### 3.5 Hypothesis 3: Mediating Effects

The third hypothesis postulated a significant indirect effect of treatment on depressive symptoms via changes in (a) avoidant attachment, (b) anxious attachment, (c) expressive suppression and (d) cognitive reappraisal. The indirect effects are reported in Table 4. All Bayes factors for the tests of the indirect effects showed strong evidence for the null hypothesis (BF_01_ = 77.37 to BF_01_ = 2668.13), indicating evidence against the proposed mediating role of anxious and avoidant attachment and expressive suppression and cognitive reappraisal in psychotherapeutic treatment for depression.

**TABLE 4:** Indirect effects.

Indirect effect	Estimate	SD_post	CI_lower	CI_upper	BF_01_
Model 1: Expressive suppression					
Mediation effects					
Treatment → T2 ES → T3 DS	9.901e−4	0.005	−0.008	0.117	1212.503
Treatment → T3 ES → T4 DS	5.618e−5	0.004	−0.012	0.013	1051.244
Reverse mediation effects					
Treatment → T2 DS → T3 ES	1.126e−2	0.009	−0.002	0.032	152.375
Treatment → T3 DS → T4 ES	1.610e−2	0.012	−0.004	0.043	77.374
Model 2: Cognitive reappraisal					
Mediation effects					
Treatment → T2 CR → T3 DS	0.001	0.004	−0.006	0.009	2668.126
Treatment → T3 CR → T4 DS	0.001	0.005	−0.009	0.014	1516.080
Reverse mediation effects					
Treatment → T2 DS → T3 CR	−0.017	0.014	−0.489	0.006	78.907
Treatment → T3 DS → T4 CR	−0.003	0.008	−0.023	0.012	674.919
Model 3: Avoidant attachment					
Mediation effects					
Treatment → T3 AV → T4 DS	−0.001	0.005	−0.013	0.010	1379.542
Reverse mediation effects					
Treatment → T3 DS → T4 AV	0.012	0.011	−0.004	0.039	164.392
Model 4: Anxious attachment					
Mediation effects					
Treatment → T3 AX → T4 DS	0.000	0.005	−0.009	0.011	1750.525
Reverse mediation effects					
Treatment → T3 DS → T4 AX	0.019	0.015	−0.003	0.055	89.204

Abbreviations: AV = avoidant attachment, AX = anxious attachment, BF_01_ = Bayes factor, CI_lower = lower bound of the 95% credible interval, CI_upper = upper bound of the 95% credible interval, CR = cognitive reappraisal, DS = depression, ES = expressive suppression, SD_post = standard deviation of posterior distribution.

### 3.6 Exploratory Analyses

To test whether our data support findings from the existing literature indicating that CBT and ST improve emotion regulation and attachment, we conducted exploratory regression analyses with treatment and time as predictors, with and without interactions. These showed a significant improvement in all proposed MoC at follow‐up (without interactions: *p* < 0.001 to *p* = 0.002, BF_10_ = 4.631 to BF_10_ = 399.684; with interactions: *p* < 0.001 to *p* = 0.019; BF_10_ = 0.545 to BF_10_ = 199.515). None of the regression analyses showed a significant effect of treatment on the MoC (*p* = 0.165 to *p* = 0.950; BF_01_ = 4.902 to BF_01_ = 10.989), but there was a significant treatment × time interaction for cognitive reappraisal at T4, β = −0.649, *p* = 0.006, BF10 = 5.215, indicating that cognitive reappraisal was lower in schema therapy at follow‐up.

## 4 Discussion

Understanding mechanisms of change (MoC) in the psychotherapeutic treatment of depression is crucial for improving treatment effectiveness to pave the way for precision psychotherapy: If ST and CBT act through different mechanisms, measures of these mechanisms at intake could inform from which treatment a patient potentially benefits more. We therefore tested specific MoC, which play a relevant role not only in depression but also in the therapy rationales of cognitive behaviour therapy (CBT) schema therapy (ST). We investigated if CBT and ST have differential effects on anxious and avoidant attachment and expressive suppression and cognitive reappraisal (H1), if changes in these MoC predict changes in depressive symptoms (H2) and if these MoC mediate treatment effects on depressive symptoms (H3). The data did not support these hypotheses but consistently supported the corresponding null hypotheses. The results add two important aspects to the current literature.

First, we could not replicate findings from previous research that emotion regulation or attachment predicted symptom change (H2). This might be mainly attributed to the fact that other studies investigated different constructs. In the case of emotion regulation (Radkovsky et al. 2014), they investigated tolerance and modification, compared to expressive suppression and cognitive reappraisal in our study, and their population received only CBT‐based interventions. Regarding attachment, Levy et al. (2018) used attachment security and Dagan et al. (2018) used insecure‐preoccupation in their meta‐analyses whereas we looked at avoidant and anxious attachment. Baljé et al. (2025) did indeed find a mediating effect of some investigated mechanisms (self‐esteem, average value of schema mode expressions and the avoidant protector mode) at mid treatment on social anxiety symptoms at post treatment. Their study resembles ours in several respects: It compares ST and CBT, and it investigates mechanisms of change including emotion regulation, but assessed via the DERS (as compared to the ERQ) subscales nonacceptance of emotional responses, difficulty engaging in goal‐directed behaviour, impulse control difficulties, limited access to emotion regulation strategies and lack of emotional clarity. It differs, however, in others—most notably, it draws on an outpatient sample with social anxiety disorder and avoidant personality disorder rather than our sample with depressive disorder. With the DERS, they measured different aspects of emotion regulation (see above) than we did with expressive suppression and cognitive reappraisal but likewise found no mediating effect. Another common finding of both studies is that neither Baljé and colleagues nor ourselves found a specific impact of the psychotherapeutic treatments on the MoC. The discrepancies of our findings in comparison with the above mentioned studies implicate that the definition of the constructs and the operationalization of the measurements need to be more specific and precise and primarily more consistent and congruent across studies. This means, for instance, that more studies should agree on and use the same constructs and measurements.

Second, in an exploratory analysis, we found significant improvements in all investigated MoC when comparing baseline and follow‐up scores. We also found reverse association effects from depressive symptoms at post‐treatment to expressive suppression at the 6‐month follow‐up, as well as from depressive symptoms at mid‐treatment to cognitive reappraisal at post‐treatment. Interestingly, another study (Quaatz et al. 2026) found the same temporal direction, with change in depressive symptoms preceding change in emotion regulation. It used a different methodology (emotion regulation and depressive symptoms operationalized as daily ecological momentary assessment items, emotion regulation measured by a single item only, ‘Are you having a hard time dealing with negative emotions right now?’) and different statistics (contemporaneous and temporal network estimation using multilevel vector autoregression). They found that better than average mood on 1 day was associated with fewer difficulties in emotion regulation on the next day. Taken together, these observations might speak rather in favour of the mechanisms as consequences of change, as opposed to MoC driving the change on a symptom level. But given the limitations of this study (see below), these findings should be replicated in future studies exploring if there are favourable mood states or phases to impact mechanisms or skills such as emotion regulation, rather than the other way round targeting emotion regulation to improve depressive symptoms.

The study has several methodological strengths enhancing the internal and external validity of our findings. Random‐intercept cross‐lagged panel models (RI‐CLPM) disentangled stable between‐person traits from the fluctuating within‐person shifts required to establish a true mechanism of change. The transition to a Bayesian framework rectified estimation errors (Heywood cases) while providing, through Bayes factors, a continuous measure of the weight of evidence for the null hypothesis. Ecological and clinical validity are also high: Investigating a severely depressed sample (*N* = 198) in an intensive inpatient and day‐clinic routine psychiatric care setting addresses an important gap in mechanism research, a field which often relies on less impaired outpatient populations. Data were moreover collected within a rigorously implemented randomized clinical trial including rater blinded manual adherence checks of the interventions delivered by the study therapists (Kopf‐Beck et al. 2020).

However, the study also has several limitations. The intensive multimodal treatment setting has been shown to have both positive and negative effects: While the concurrent application of pharmacotherapy, individual/group psychotherapy and occupational therapy is representative of ‘real‐world’ psychiatric care, it may have obscured the differential effects of CBT versus schema therapy and the specific effects of psychotherapy. Another limitation pertains to construct specificity: We used the Emotion Regulation Questionnaire (ERQ), which is oriented towards the specific strategies of cognitive reappraisal and expressive suppression, whereas, as discussed above, other research findings suggest that other constructs may hold higher prognostic value. Also, all our findings apply to our inpatient and day clinic sample of patients with acute depression expressed in a severe symptomatology; it is not clear if other dynamics of MoC apply in other (less burdened) samples. Finally, two design limitations should be mentioned: First, temporal aspects: The intervals between measurements were unevenly distributed and anxious and avoidant attachment were not measured at T2. The availability and temporal resolution of the data might therefore not have detected changes of the MoC under investigation, since these might operate at different temporal dynamics. Additionally, 7 weeks of treatment might not allow enough time for the treatments, specifically for the experiential ST techniques to have an impact on the MoC and/or for the changes in the MoC to unfold. Second, our sample is relatively small given the complexity of the RI‐CLPM and could have led to the negative variances (Heywood cases) in the original RI‐CLPM; our trouble shooting simulation with *N* = 2000 cases (Appendix S2) shows that *N* could have been one factor here. While the Bayesian parameter estimation methods did not suffer from the same convergence problems, the statements regarding the evidence supporting the null hypothesis should still be made with some caution as they depend on the exact model specification of the complex RI‐CLPM.

## 5 Conclusions

In contrast to the predictive mechanics observed in outpatient populations, the temporal precedence identified in this inpatient and day clinic sample suggests that emotion regulatory shifts are consequences of changes in depressive symptoms rather than their primary drivers. We suggest exploring in further studies if mood improvement may create necessary mood states or phases for subsequent therapeutic work on the mechanisms to consolidate skills. Moreover, we could not find differential effects between Schema Therapy and Cognitive Behaviour Therapy. This is consistent with findings in the field which most often fail to find differential effects between psychotherapeutic interventions. In future studies, the further identification of temporal dynamics could advance precision psychotherapy by guiding the gaze of clinicians and researchers from what specific interventions to do towards when to do them.

## Author Contributions

All authors contributed to conceptualization, analysis, writing and revising the manuscript. Theresa Badenbach and Angelika Stefan executed the statistical analysis. Samy Egli, Arnold van Emmerik, Angelika Stefan and Johannes Kopf‐Beck supervised the project. Members of the OPTIMA Study Group contributed to conceptualization, data collection and data preprocessing.

## Funding

The original OPTIMA trial was funded by the Max Planck Society. The funder had no role in the design, data collection, data analysis and reporting of this study. The current secondary analysis was not supported by any sponsor or funder.

## Ethics Statement

The original OPTIMA trial is registered at clinicaltrials.gov (NCT03287362). It was approved by the Institutional Ethic Committee of the Faculty of Medicine of Ludwig‐Maximilians‐University Munich (project number 17‐395). Modifications of the protocol were reported to the committee. All participants were informed in oral and written form about the study, treatments, assessments and duration of the study. Written informed consent was obligatory prior to enrolment.

## Conflicts of Interest

The authors declare no conflicts of interest.

## Supporting information


**Appendix S1:** Results of the cross‐lagged panel models without random intercepts.

**Appendix S2:** Troubleshooting procedure for the Heywood cases in the RI‐CLPM estimations.

## Data Availability

The datasets generated and/or analysed for the current study contain clinical data and are not publicly available due to the protection of participants' rights to privacy and data protection but are available from the corresponding author on reasonable request.

## References

[cpp70345-bib-0001] Abler, B. , and H. Kessler . 2009. “Emotion Regulation Questionnaire—Eine deutschsprachige Fassung des ERQ von Gross und John.” Diagnostica 55, no. 3: 144–152. 10.1026/0012-1924.55.3.144.

[cpp70345-bib-0002] Baljé, A. E. , A. Greeven , M. Deen , A. E. van Giezen , A. Arntz , and P. Spinhoven . 2025. “Exploring Moderators and Mediators of the Outcome of Group Cognitive Behavioural Therapy Compared With Group Schema Therapy for Social Anxiety Disorder and Comorbid Avoidant Personality Disorder.” Clinical Psychology & Psychotherapy 32, no. 5: e70148. 10.1002/cpp.70148. PMC12516937 41081422

[cpp70345-bib-0003] Banyard, H. , A. J. Behn , and J. Delgadillo . 2021. “Personality Disorders and Their Relation to Treatment Outcomes in Cognitive Behavioural Therapy for Depression: A Systematic Review and Meta‐Analysis.” Cognitive Therapy and Research 45, no. 4: 561–576. 10.1007/s10608-021-10203-x.

[cpp70345-bib-0004] Berking, M. , D. Ebert , P. Cuijpers , and S. G. Hofmann . 2013. “Emotion Regulation Skills Training Enhances the Efficacy of Inpatient Cognitive Behavioral Therapy for Major Depressive Disorder: A Randomized Controlled Trial.” Psychotherapy and Psychosomatics 82, no. 4: 234–245. 10.1159/000348448. 23712210

[cpp70345-bib-0005] Bernecker, S. L. , M. J. Constantino , L. R. Atkinson , R. M. Bagby , P. Ravitz , and C. McBride . 2016. “Attachment Style as a Moderating Influence on the Efficacy of Cognitive‐Behavioral and Interpersonal Psychotherapy for Depression: A Failure to Replicate.” Psychotherapy 53, no. 1: 22–33. 10.1037/pst0000036. 26726822

[cpp70345-bib-0006] Bowlby, J. 1969. Attachment and Loss: Attachment (Bd. 1). Basic Books.

[cpp70345-bib-0007] Bowlby, J. 1980. Attachment and Loss: Sadness and Depression (Bd. 3). Basic Books.

[cpp70345-bib-0008] Bowlby, J. 1988. A Secure Base: Clinical Applications of Attachment Theory. 1st ed. Routledge.

[cpp70345-bib-0009] Carter, J. D. , V. V. McIntosh , J. Jordan , R. J. Porter , C. M. Frampton , and P. R. Joyce . 2013. “Psychotherapy for Depression: A Randomized Clinical Trial Comparing Schema Therapy and Cognitive Behavior Therapy.” Journal of Affective Disorders 151, no. 2: 500–505. 10.1016/j.jad.2013.06.034. 23870427

[cpp70345-bib-0010] Clark, D. A. 2022. “Cognitive Reappraisal.” Cognitive and Behavioral Practice 29, no. 3: 564–566. 10.1016/j.cbpra.2022.02.018.

[cpp70345-bib-0011] Conradi, H. J. , J. H. Kamphuis , and P. De Jonge . 2018. “Adult Attachment Predicts the Seven‐Year Course of Recurrent Depression in Primary Care.” Journal of Affective Disorders 225: 160–166. 10.1016/j.jad.2017.08.009. 28837949

[cpp70345-bib-0012] Cuijpers, P. , C. Miguel , M. Harrer , et al. 2023. “Cognitive Behavior Therapy vs. Control Conditions, Other Psychotherapies, Pharmacotherapies and Combined Treatment for Depression: A Comprehensive Meta‐Analysis Including 409 Trials With 52,702 Patients.” World Psychiatry 22, no. 1: 105–115. 10.1002/wps.21069. PMC9840507 36640411

[cpp70345-bib-0013] Dagan, O. , C. R. Facompré , and K. Bernard . 2018. “Adult Attachment Representations and Depressive Symptoms: A Meta‐Analysis.” Journal of Affective Disorders 236: 274–290. 10.1016/j.jad.2018.04.091. 29751243

[cpp70345-bib-0014] Egli, S. , E. Frieß , P. Graf , et al. 2019. Schematherapie bei Depressionen: Ein Behandlungskonzept für das (teil)stationäre Setting. Hogrefe Verlag.

[cpp70345-bib-0015] Falkenström, F. , N. Solomonov , and J. A. Rubel . 2022. “How to Model and Interpret Cross‐Lagged Effects in Psychotherapy Mechanisms of Change Research: A Comparison of Multilevel and Structural Equation Models.” Journal of Consulting and Clinical Psychology 90, no. 5: 446–458. 10.1037/ccp0000727. PMC9245087 35604748

[cpp70345-bib-0016] Farooq, R. 2022. “Heywood Cases: Possible Causes and Solutions.” International Journal of Data Analysis Techniques and Strategies 14, no. 1: 79–88. 10.1504/IJDATS.2022.121506.

[cpp70345-bib-0017] Fassbinder, E. , U. Schweiger , D. Martius , O. Brand‐de Wilde , and A. Arntz . 2016. “Emotion Regulation in Schema Therapy and Dialectical Behavior Therapy.” Frontiers in Psychology 7. 10.3389/fpsyg.2016.01373 PMC5021701 27683567

[cpp70345-bib-0018] Forkmann, T. , A. Scherer , M. Pawelzik , et al. 2014. “Does Cognitive Behavior Therapy Alter Emotion Regulation in Inpatients With a Depressive Disorder?” Psychology Research and Behavior Management: 147. 10.2147/PRBM.S59421. PMC4026562 24872725

[cpp70345-bib-0019] Gratz, K. L. , and L. Roemer . 2004. “Multidimensional Assessment of Emotion Regulation and Dysregulation: Development, Factor Structure, and Initial Validation of the Difficulties in Emotion Regulation Scale.” Journal of Psychopathology and Behavioral Assessment 26, no. 1: 41–54. 10.1023/B:JOBA.0000007455.08539.94.

[cpp70345-bib-0020] Hamaker, E. L. , R. M. Kuiper , and R. P. P. P. Grasman . 2015. “A Critique of the Cross‐Lagged Panel Model.” Psychological Methods 20, no. 1: 102–116. 10.1037/a0038889. 25822208

[cpp70345-bib-0021] Harris, D. 2010. “Inhaltliche Validierung der deutschen Übersetzung des Schema Mode Inventory—Revised (SMI‐r) und Korrelation der Modus‐ Skalen des SMI‐r mit etablierten psychologischen und psychopathologischen Konstrukten.”

[cpp70345-bib-0022] Hautzinger, M. 2013. Kognitive Verhaltenstherapie bei Depressionen: Mit Online‐Materialien. 7th, vollständig überarbeitete und erweiterte ed. Beltz.

[cpp70345-bib-0023] Hautzinger, M. , F. Keller , C. Kühner , and A. T. Beck . 2009. Beck‐Depressions‐Inventar: BDI II; Revision; Manual. 2nd ed. Pearson.

[cpp70345-bib-0024] Hoffmann, T. C. , P. P. Glasziou , I. Boutron , et al. 2014. “Better Reporting of Interventions: Template for Intervention Description and Replication (TIDieR) Checklist and Guide.” BMJ 348: g1687. 10.1136/bmj.g1687. 24609605

[cpp70345-bib-0025] Hohls, J. K. , H.‐H. König , E. Quirke , and A. Hajek . 2021. “Anxiety, Depression and Quality of Life—A Systematic Review of Evidence From Longitudinal Observational Studies.” International Journal of Environmental Research and Public Health 18, no. 22: 12022. 10.3390/ijerph182212022. PMC8621394 34831779

[cpp70345-bib-0026] Jak, S. , T. D. Jorgensen , M. G. E. Verdam , F. J. Oort , and L. Elffers . 2021. “Analytical Power Calculations for Structural Equation Modeling: A Tutorial and Shiny App.” Behavior Research Methods 53, no. 4: 1385–1406. 10.3758/s13428-020-01479-0. PMC8367885 33140375

[cpp70345-bib-0027] Jorgensen, T. D. , S. Pornprasertmanit , A. M. Schoemann , et al. 2022. “semTools: Useful Tools for Structural Equation Modeling.” https://cran.r‐project.org/web/packages/semTools/index.html.

[cpp70345-bib-0028] Kazdin, A. E. 2007. “Mediators and Mechanisms of Change in Psychotherapy Research.” Annual Review of Clinical Psychology 3, no. 1: 1–27. 10.1146/annurev.clinpsy.3.022806.091432. 17716046

[cpp70345-bib-0029] König, H. , H.‐H. König , and A. Konnopka . 2020. “The Excess Costs of Depression: A Systematic Review and Meta‐Analysis.” Epidemiology and Psychiatric Sciences 29: e30. 10.1017/S2045796019000180. PMC8061284 30947759

[cpp70345-bib-0030] Kool, M. , H. Van , A. Arntz , et al. 2024. “Dosage Effects of Psychodynamic and Schema Therapy in People With Comorbid Depression and Personality Disorder: Four‐Arm Pragmatic Randomised Controlled Trial.” British Journal of Psychiatry 225, no. 1: 274–281. 10.1192/bjp.2024.56. 38602168

[cpp70345-bib-0031] Kopf‐Beck, J. , C. L. Müller , J. Tamm , et al. 2024. “Effectiveness of Schema Therapy Versus Cognitive Behavioral Therapy Versus Supportive Therapy for Depression in Inpatient and Day Clinic Settings: A Randomized Clinical Trial.” Psychotherapy and Psychosomatics: 1–12. 10.1159/000535492. PMC10880804 38176391

[cpp70345-bib-0032] Kopf‐Beck, J. , P. Zimmermann , S. Egli , et al. 2020. “Schema Therapy Versus Cognitive Behavioral Therapy Versus Individual Supportive Therapy for Depression in an Inpatient and Day Clinic Setting: Study Protocol of the OPTIMA‐RCT.” BMC Psychiatry 20, no. 1: 506. 10.1186/s12888-020-02880-x. PMC7557007 33054737

[cpp70345-bib-0033] Kriston, L. , J. Schäfer , G. A. Jacob , M. Härter , and L. P. Hölzel . 2013. “Reliability and Validity of the German Version of the Young Schema Questionnaire—Short Form 3 (YSQ‐S3).” European Journal of Psychological Assessment 29, no. 3: 205–212. 10.1027/1015-5759/a000143.

[cpp70345-bib-0034] Kühner, C. , C. Bürger , F. Keller , and M. Hautzinger . 2007. “Reliabilität und Validität des revidierten Beck‐Depressionsinventars (BDI‐II).” Der Nervenarzt 78, no. 6: 651–656. 10.1007/s00115-006-2098-7. 16832698

[cpp70345-bib-0035] Lange, J. , S. Goerigk , K. Nowak , R. Rosner , and A. Erhardt . 2021. “Attachment Style Change and Working Alliance in Panic Disorder Patients Treated With Cognitive Behavioral Therapy.” Psychotherapy 58, no. 2: 206–218. 10.1037/pst0000365. 34410790

[cpp70345-bib-0036] Levy, K. N. , Y. Kivity , B. N. Johnson , and C. V. Gooch . 2018. “Adult Attachment as a Predictor and Moderator of Psychotherapy Outcome: A Meta‐Analysis.” Journal of Clinical Psychology 74, no. 11: 1996–2013. 10.1002/jclp.22685. 30238450

[cpp70345-bib-0037] Liu, D. Y. , and R. J. Thompson . 2017. “Selection and Implementation of Emotion Regulation Strategies in Major Depressive Disorder: An Integrative Review.” Clinical Psychology Review 57: 183–194. 10.1016/j.cpr.2017.07.004. 28739277

[cpp70345-bib-0038] Lorenzo‐Luaces, L. , J. R. Keefe , and R. J. DeRubeis . 2016. “Cognitive‐Behavioral Therapy: Nature and Relation to Non‐Cognitive Behavioral Therapy.” Behavior Therapy 47, no. 6: 785–803. 10.1016/j.beth.2016.02.012. 27993333

[cpp70345-bib-0039] Martin, J. K. , and R. P. McDonald . 1975. “Bayesian Estimation in Unrestricted Factor Analysis: A Treatment for Heywood Cases.” Psychometrika 40, no. 4: 505–517. 10.1007/BF02291552.

[cpp70345-bib-0040] McBride, C. , L. Atkinson , L. C. Quilty , and R. M. Bagby . 2006. “Attachment as Moderator of Treatment Outcome in Major Depression: A Randomized Control Trial of Interpersonal Psychotherapy Versus Cognitive Behavior Therapy.” Journal of Consulting and Clinical Psychology 74, no. 6: 1041–1054. 10.1037/0022-006X.74.6.1041. 17154734

[cpp70345-bib-0041] Mulder, J. D. , and E. L. Hamaker . 2021. “Three Extensions of the Random Intercept Cross‐Lagged Panel Model.” Structural Equation Modeling: A Multidisciplinary Journal 28, no. 4: 638–648. 10.1080/10705511.2020.1784738.

[cpp70345-bib-0042] Nanni, V. , R. Uher , and A. Danese . 2012. “Childhood Maltreatment Predicts Unfavorable Course of Illness and Treatment Outcome in Depression: A Meta‐Analysis.” American Journal of Psychiatry 169: 141–151. 10.1176/appi.ajp.2011.11020335. 22420036

[cpp70345-bib-0043] National Institute for Health and Care Excellence . 2022. “Depression in Adults: Treatment and Management.” 35977056

[cpp70345-bib-0044] Nelson, J. , A. Klumparendt , P. Doebler , and T. Ehring . 2017. “Childhood Maltreatment and Characteristics of Adult Depression: Meta‐Analysis.” British Journal of Psychiatry: The Journal of Mental Science 210, no. 2: 96–104. 10.1192/bjp.bp.115.180752. 27908895

[cpp70345-bib-0045] Newton‐Howes, G. , P. Tyrer , T. Johnson , et al. 2014. “Influence of Personality on the Outcome of Treatment in Depression: Systematic Review and Meta‐Analysis.” Journal of Personality Disorders 28, no. 4: 577–593. 10.1521/pedi_2013_27_070. 24256103

[cpp70345-bib-0046] Quaatz, S. M. , S. Benz , L. Just , et al. 2026. “Symptom Networks in Depression: Temporal Dynamics of Mood, Cognition, and Behavior During Treatment.” Cognitive Therapy and Research 50: 867–883. 10.1007/s10608-025-10709-8.

[cpp70345-bib-0047] Quigley , D. J. A. Dozois , R. M. Bagby , D. S. S. Lobo , L. Ravindran , and L. C. Quilty . 2018. “Cognitive Change in Cognitive‐Behavioural Therapy v. Pharmacotherapy for Adult Depression: A Longitudinal Mediation Analysis.” Psychological Medicine 49. 10.1017/S0033291718003653 30560738

[cpp70345-bib-0048] Radkovsky, A. , J. J. McArdle , C. L. H. Bockting , and M. Berking . 2014. “Successful Emotion Regulation Skills Application Predicts Subsequent Reduction of Symptom Severity During Treatment of Major Depressive Disorder.” Journal of Consulting and Clinical Psychology 82, no. 2: 248–262. 10.1037/a0035828. 24564219

[cpp70345-bib-0049] Ratzsch, J. , M. Richter , R. Blitz , et al. 2025. “Childhood Maltreatment's Influence on the Dynamic Course of Depression: Symptom Trajectories During Inpatient Treatment and After Discharge.” Psychological Medicine 55: e127. 10.1017/S0033291725000984. PMC12094660 40313139

[cpp70345-bib-0050] Renner, F. , A. Arntz , I. Leeuw , and M. Huibers . 2013. “Treatment for Chronic Depression Using Schema Therapy.” Clinical Psychology: Science and Practice 20, no. 2: 166–180. 10.1111/cpsp.12032.

[cpp70345-bib-0051] Rottenberg, J. 2017. “Emotions in Depression: What Do We Really Know?” Annual Review of Clinical Psychology 13, no. 1: 241–263. 10.1146/annurev-clinpsy-032816-045252. 28375721

[cpp70345-bib-0052] Satorra, A. , and W. E. Saris . 1985. “Power of the Likelihood Ratio Test in Covariance Structure Analysis.” Psychometrika 50, no. 1: 83–90. 10.1007/BF02294150.

[cpp70345-bib-0053] Sloan, E. , K. Hall , R. Moulding , S. Bryce , H. Mildred , and P. K. Staiger . 2017. “Emotion Regulation as a Transdiagnostic Treatment Construct Across Anxiety, Depression, Substance, Eating and Borderline Personality Disorders: A Systematic Review.” Clinical Psychology Review 57: 141–163. 10.1016/j.cpr.2017.09.002. 28941927

[cpp70345-bib-0054] Steffanowski, A. , M. Oppl , M. Meyerberg , J. Schmidt , W. Wittmann , and R. Nübling . 2001. “Psychometrische Überprüfung einer deutschsprachigen Version des Relationship Scales Questionaire (RSQ).” In Störungsspezifische Therapieansätze—Konzepte und Ergebnisse, 320–342. Psychosozial.

[cpp70345-bib-0055] Taylor, P. , J. Rietzschel , A. Danquah , and K. Berry . 2015. “Changes in Attachment Representations During Psychological Therapy.” Psychotherapy Research 25, no. 2: 222–238. 10.1080/10503307.2014.886791. 24559454

[cpp70345-bib-0056] van Doorn, J. , D. van den Bergh , U. Böhm , et al. 2021. “The JASP Guidelines for Conducting and Reporting a Bayesian Analysis.” Psychonomic Bulletin & Review 28, no. 3: 813–826. 10.3758/s13423-020-01798-5. PMC8219590 33037582

[cpp70345-bib-0057] Visted, E. , J. Vøllestad , M. B. Nielsen , and E. Schanche . 2018. “Emotion Regulation in Current and Remitted Depression: A Systematic Review and Meta‐Analysis.” Frontiers in Psychology 9. 10.3389/fpsyg.2018.00756. PMC5968125 29867700

[cpp70345-bib-0058] World Health Organization . 2023. “Depressive Disorder (Depression).” https://www.who.int/news‐room/fact‐sheets/detail/depression.

[cpp70345-bib-0059] Yakın, D. , T. Gençöz , L. Steenbergen , and A. Arntz . 2019. “An Integrative Perspective on the Interplay Between Early Maladaptive Schemas and Mental Health: The Role of Self‐Compassion and Emotion Regulation.” Journal of Clinical Psychology 75, no. 6: 1098–1113. 10.1002/jclp.22755. PMC6593850 30735580

[cpp70345-bib-0060] Young, J. E. , J. S. Klosko , and M. E. Weishaar . 2003. Schema Therapy: A Practitioner's Guide (S. 436). Guilford Press.

[cpp70345-bib-0061] Zyphur, M. J. , P. D. Allison , L. Tay , et al. 2020. “From Data to Causes I: Building a General Cross‐Lagged Panel Model (GCLM).” Organizational Research Methods 23, no. 4: 651–687. 10.1177/1094428119847278.

