# Supplementary material for: Mechanisms of Change in the Treatment of Depression: On the Role of Attachment, Reappraisal and Suppression
Source: Clin Psychol Psychother. 2026 Sep 30;33(5):e70345. doi: 10.1002/cpp.70345 (PMC13626740; doi:10.1002/cpp.70345)
Supplement: Supplementary file 1 [file CPP-33-e70345-s001.docx]

**Appendix B: Troubleshooting procedure for the Heywood cases in the RI-CLPM estimations**

In structural equation models, estimates can take on values that are impossible for the corresponding parameter in the population (Bollen, 1987). This is referred to as improper solutions. One instance of an improper solution are negative variances, so-called Heywood cases (Chen, Bollen, Paxton, Curran, & Kirby, 2001). Heywood cases can occur for several reasons such as underidentification, non-convergence, outliers, small sample size, missing values, sampling fluctuations, empirical underidentification, or model misspecification (Chen et al., 2001; Farooq, 2022; Newsom, Hachem, Granger, & Denning, 2023). In the present study, Heywood cases occurred in the estimation of four random intercept cross-lagged panel models (RI-CLPMs) to investigate attachment and emotion regulation as mechanisms of change in schema therapy versus cognitive behaviour therapy for depression. This appendix describes the steps conducted for diagnosing the cause of the Heywood cases in the estimated models.

In the following, the suggestions by previous authors are synthesised (Chen et al., 2001; Farooq, 2022; Newsom et al., 2023) and the practical steps for investigating the possible causes for Heywood cases are described. As the problem occurred in all four models and the same procedure was followed for all of them, the steps are described using the RI-CLPM for emotion suppression and depressive symptoms as an example. All steps were conducted in R using the lavaan package (Rosseel, 2012).

First, Heywood cases are more likely when a variable has a very different variance than the rest of the variables in the sample (Yorgason, 2014). As depressive symptoms were measured on a scale from 1-64, attachment on a scale from 1-5, and emotion regulation on a scale from 1-7, the variances (i.e., the standard deviations) differed substantially in the present data. To address this problem, all variables were normalised by rescaling them to a scale from 0 to 1 (Yorgason, 2014). However, this step did not solve the negative variances.

Second, as suggested by Chen et al. (2001), the model identification was checked. A structural equation model is identified when it has nonnegative degrees of freedom, which means that the number of free parameters is equal to or lower than the number of known values in the variance-covariance matrix (Kline, 2016). When a model is underidentified, it cannot be estimated. Estimating the unconstrained model with all parameters allowed to vary freely led to a model with df = 18, indicating that the model is identified.

Third, Chen et al. (2001) suggest estimating the unconstrained model and determining whether the model converges to a solution. The present model converged, but a negative variance occurred for the variance of the random intercept of depressive symptoms (estimate = -.018; p >.05).

Fourth, Chen et al. (2001) recommend checking the data for outliers. Univariate outliers were evaluated through histograms and boxplots and two outliers were found for depressive symptoms at the second measurement wave. Re-estimating the model after outlier removal did not qualitatively change the model estimates. A further removal of two participants who completed the questionnaire at only one measurement wave also had no influence on the results.

Fifth, univariate and multivariate normality was evaluated through Q-Q plots. The plots indicated that the assumption of multivariate normality was violated. Since the initially used maximum likelihood (ML) estimation method assumes multivariate normality, the model fits were repeated using robust ML estimation that can handle non-normal data. Two robust options are available in lavaan for data with missing values: MLF for a conventional test statistic and robust standard errors based on the first order derivates, and MLR for a scaled test statistic with robust (Huber-White) standard errors (Rosseel, 2012). However, the negative variance prevailed under re-estimation with both robust estimators.

Sixth, if negative variances are not due to outliers, Chen et al. (2001) suggest checking for empirical underidentification. This refers to the situation that the model has positive degrees of freedom, but there is insufficient covariance information in a portion of the model for the software to generate valid estimates (Newsom et al., 2023). Empirical underidentification can result from scaling problems or high multicollinearity (Newsom et al., 2023). Scaling problems are present when insufficient factor loadings are constrained to 1, but this was not the case in the present model. Multicollinearity was evaluated through the variance inflation factor (VIF; see e.g., Marcoulides and Raykov, (2019). All VIFs were below 5, which is a frequently suggested threshold for acceptable multicollinearity (Marcoulides & Raykov, 2019), indicating that high multicollinearity was also not the reason for the Heywood cases.

Seventh, we tested whether the negative variances are due to sampling fluctuations. This is done by comparing the unconstrained model with a constrained model that sets the negative variance to zero or a small positive value. If the tests suggests that the variance is below zero, indicated by a significant test result, model misspecification should be suspected. A nonsignificant test result indicates that the negative variances are due to sampling fluctuations. In this case, Chen and colleagues (2001) suggest to re-estimate the model with the negative variance set to zero. For our models, the likelihood ratio test was nonsignificant (*p* > .05). However, re-estimating the model with the variances constrained to zero led to a non-positive definite covariance matrix. According to Newsom et al. (2023) this frequently occurs due to empirical underidentification or due to a high proportion of missing values. Since the data had substantial proportion of missing values (22.8%), two strategies were employed next to check whether these were problematic. First, the model was re-estimated with multiple imputation handling missing values instead of robust ML estimation. The mice package (Buuren & Groothuis-Oudshoorn, 2011) was used to impute the missing values. Second, the model was re-estimated without the fourth measurement wave, as this measurement wave had the highest proportion of missing values (i.e., 57% missing values for emotion suppression at T4). However, both strategies did not prevent the occurrence of Heywood cases.

Eighth, we imposed equality constraints between parameters to reduce model complexity and address potential underidentification problems (Farooq, 2022). Since there were unequal time lags between the measurement waves, it is theoretically implausible to assume equal cross-lagged effects. However, as the residual variances of the observed variables are not of primary interest, the model was re-estimated with the residual variances constrained to be equal. This step imposes the assumption of time-invariant residual variances. However, the constrained model still displayed negative variances for the random intercept of depressive symptoms.

Finally, we simulated data to understand under which circumstances the unconstrained RI-CLPM can be fitted properly. When simulating data from a multivariate normal distribution with *N* = 2000, the same variance-covariance matrix and means as the observed data, and no missing values, the model could be fitted without negative variances. However, when fitting the model with the same properties but *N* = 200 as in the observed data, it led to negative variances. While it is not possible to isolate whether the larger sample alone or the combination with a multivariate normal distribution and the absence of missing values led the model to fit, this shows that it is in principle possible to fit a RI-CLPM on data with the observed means and covariance structures.

In sum, it remains difficult to conclusively say what caused the Heywood cases in the present situation despite extensive troubleshooting. The most likely reason for these Heywood cases was a combination of missing data and low sample size in comparison to the complexity of the model. Various solutions suggested in past literature, such as constraining the problematic variances to zero, constraining similar parameter estimates to be equal, and reducing missing values by excluding the last measurement wave failed to address the problem. Obtaining a larger sample may be a solution, but this was not feasible in the present study. Since none of the adjustments to the preregistered analyses were successful at removing the Heywood cases, we chose to switch to a Bayesian analysis framework instead to answer the research questions. The Bayesian analysis framework allows for hard constraints on parameter values through the specification of adequate prior distributions and thus makes negative variance estimates impossible. Switching from a maximum likelihood estimation to a Bayesian estimation framework has been suggested before as a solution for dealing with Heywood cases(Martin & McDonald, 1975). In our case, it allowed us to stay as close as possible to the preregistration and to maintain the benefits of the random intercept model. An alternative approach (reducing the model to a simple cross-lagged panel model without random intercepts) is reported in appendix A. The results of the Bayesian analyses are reported in the main paper.

**References**

Bollen, K. A. (1987). Outliers and Improper Solutions: A Confirmatory Factor Analysis Example. *Sociological Methods & Research*, *15*(4), 375–384. doi: 10.1177/0049124187015004002

Buuren, S. van, & Groothuis-Oudshoorn, K. (2011). mice: Multivariate Imputation by Chained Equations in R. *Journal of Statistical Software*, *45*, 1–67. doi: 10.18637/jss.v045.i03

Chen, F., Bollen, K. A., Paxton, P., Curran, P. J., & Kirby, J. B. (2001). Improper Solutions in Structural Equation Models: Causes, Consequences, and Strategies. *Sociological Methods & Research*, *29*(4), 468–508. doi: 10.1177/0049124101029004003

Farooq, R. (2022). Heywood cases: Possible causes and solutions. *International Journal of Data Analysis Techniques and Strategies*, *14*(1), 79–88. doi: 10.1504/IJDATS.2022.121506

Kline, R. B. (2016). *Principles and practice of structural equation modeling, 4th ed* (pp. xvii, 534). New York, NY, US: Guilford Press.

Marcoulides, K. M., & Raykov, T. (2019). Evaluation of Variance Inflation Factors in Regression Models Using Latent Variable Modeling Methods. *Educational and Psychological Measurement*, *79*(5), 874–882. doi: 10.1177/0013164418817803

Martin, J. K., & McDonald, R. P. (1975). Bayesian estimation in unrestricted factor analysis: A treatment for heywood cases. *Psychometrika*, *40*(4), 505–517. doi: 10.1007/BF02291552

Newsom, J. T., Hachem, Z. A., Granger, A. M., & Denning, E. C. (2023). Where Did I Go Wrong with My Model? Ten Tips for Getting Results in SEM. *Structural Equation Modeling: A Multidisciplinary Journal*, *30*(3), 491–500. doi: 10.1080/10705511.2022.2101113

Rosseel, Y. (2012). lavaan: An R Package for Structural Equation Modeling. *Journal of Statistical Software*, *48*, 1–36. doi: 10.18637/jss.v048.i02

Yorgason, J. (2014). *Troubleshooting problems with SEM models that have “Heywood” cases such as negative variance parameters and non-positive definite covariance matrices*. Brigham Young University, Utah.
