# Supplementary material for: Mechanisms of Change in the Treatment of Depression: On the Role of Attachment, Reappraisal and Suppression
Source: Clin Psychol Psychother. 2026 Sep 30;33(5):e70345. doi: 10.1002/cpp.70345 (PMC13626740; doi:10.1002/cpp.70345)
Supplement: Supplementary file 2 [file CPP-33-e70345-s002.docx]

**Appendix A: Results of the Cross-Lagged Panel Models without Random Intercepts**

As the preregistered maximum-likelihood estimation of the Random Intercept Cross-Legged Panel Models (RI-CLPMs) led to Heywood cases, we used a Bayesian analysis framework instead. An alternative would have been to simplify the models to a Cross-Lagged Panel Model (CLPM) without random intercept. However, this would have come at the cost of potential confounding of between- and within-person variability (Hamaker, Kuiper, & Grasman, 2015). Below, we report the results of the CLPM analyses as robustness checks.

***Likelihood Ratio Test***

First, the unconstrained models with all parameters estimated freely were compared against the constrained models with the cross-lagged and autoregressive parameters constrained to be equal across treatment conditions through likelihood ratio tests. All likelihood ratio tests were non-significant (*p* > .05), which indicated that the cross-lagged and autoregressive effects did not differ significantly between the treatment conditions and thereby justifies the approach of modelling treatment condition as a two-level predictor. This is consistent with our findings from the Bayesian RI-CLPM.

***Cross-lagged panel models***

The fit indices for the constrained cross-lagged panel models are reported in Table A1. Good model fit indicates that the hypothesised relations in the model fit well with the observed relations in the data. The chi-square tests of all models were significant, indicating poor model fit. For anxious attachment, the p-value of the chi-square test was particularly high compared to the degrees of freedom, suggesting substantial misfit. However, the chi-square test is sensitive to sample size, and in small samples it tends to reject true models at higher rates. Since the sample size in our study was curbed by substantial missingness at later measurement waves, model fit needs to be evaluated in conjunction with other fit indices. For emotion suppression and cognitive reappraisal, all other fit indices indicated good fit. Therefore, the fit of these models was deemed acceptable. For avoidant attachment and anxious attachment, the other fit indices indicated bad fit. Specifically, both models had RMSEA values above the typical cut-off value of 0.06 (Hu & Bentler, 1999). Furthermore, the anxious attachment model additionally had an extremely low CFI value (CFI = .56). Therefore, modification indices for these models were inspected. For avoidant attachment, the highest theoretically plausible modification index suggested adding a covariance between avoidant attachment at T3 and T4. Implementing this modification led to substantially improved model fit ($\chi^{2}$ = 5.23; p = 0.39; df = 5; CFI = .99; RMSEA = .01; SRMR = .03). For anxious attachment, the two highest modification indices suggested to add covariances between anxious attachment at T3 and T4, and T1 and T4. After adding these covariances, the model fit the data well ($\chi^{2}$ = 3.58; p = 0.47; df = 4; CFI = 1; RMSEA = .00; SRMR = .03).

***Treatment effects***

The first hypothesis posed that ST would lead to greater reductions in a) avoidant attachment, b) anxious attachment, and c) emotion suppression compared to CBT, while CBT would predict greater increases in d) cognitive reappraisal compared to ST. We evaluated this hypothesis by testing the path coefficients from treatment to the mechanisms against zero. We found that CBT predicted greater increases in cognitive reappraisal than ST at the 6-month follow up measure, estimate = -0.079, *p* = .039. However, after applying the Benjamini-Hochberg^[1]^ correction for multiple testing (Benjamini & Hochberg, 1995), this effect was no longer significant, *p* = .076. No other treatment effects on the mechanisms were significant in the uncorrected and Benjamini-Hochberg corrected models. The results of the cross-lagged models after correction for multiple testing are shown in Figure A1. The non-significant results are in line with the results from the Bayesian RI-CLPM that indicate evidence for the null hypothesis.

***Cross-lagged effects***

Hypothesis 2 stated that improvements in a) avoidant attachment, b) anxious attachment, c) emotion regulation, and d) cognitive reappraisal will predict subsequent symptom improvement, providing evidence for their causal role in therapeutic change. We evaluated this hypothesis by testing the path coefficients for cross-lagged effects from the mechanisms to the depressive symptoms against zero. We found that higher levels of emotion suppression at baseline predicted more depressive symptoms at the second measurement wave, estimate = 0.184, *p* = .000. This is in contrast to the Bayesian RI-CLPM where we found moderate evidence for the null hypothesis for this particular path (BF_10_ =0.201). The other coefficient estimates did not significantly differ from zero (cf. Figure A1a), which is in line with the Bayesian RI-CLPM results. Considering reverse associations, higher levels of depressive symptoms at post-treatment predicted more emotion suppression at the 6-month follow-up (cf. Figure A1a), estimate = 0.511, *p* = .000. Furthermore, we found that lower levels of depressive symptoms at mid-treatment predicted lower levels of cognitive reappraisal at post-treatment, estimate = -0.304,  *p* = .009. All other cross-lagged effects were non-significant. This is in line with the results from the Bayesian RI-CLPM where we found evidence for the alternative for these same two paths and evidence for the null hypothesis for all others.

***Indirect effects***

The third hypothesis was that there would be a significant indirect effect of treatment on depressive symptoms via changes in the a) avoidant attachment, b) anxious attachment, c) emotion suppression and d) cognitive reappraisal. To evaluate this hypothesis, we tested the indirect paths from treatment via the mechanisms to depressive symptoms against zero. The indirect effects are reported in Table A2. None of the computed indirect paths were significant. This is in line with the Bayesian RI-CLPM results that showed extreme evidence for the null hypothesis for all mediating effects, indicating evidence against the proposed mediating role of attachment styles and emotion regulation strategies in psychotherapeutic treatment for depression.

**References**

Benjamini, Y., & Hochberg, Y. (1995). Controlling the False Discovery Rate: A Practical and Powerful Approach to Multiple Testing. *Journal of the Royal Statistical Society: Series B (Methodological)*, *57*(1), 289–300. doi: 10.1111/j.2517-6161.1995.tb02031.x

Hamaker, E. L., Kuiper, R. M., & Grasman, R. P. P. P. (2015). A critique of the cross-lagged panel model. *Psychological Methods*, *20*(1), 102–116. doi: 10.1037/a0038889

Hu, L., & Bentler, P. M. (1999). Cutoff criteria for fit indexes in covariance structure analysis: Conventional criteria versus new alternatives. *Structural Equation Modeling*, *6*(1), 1–55. doi: 10.1080/10705519909540118

**Tables and Figures**

**Table A1**

*Goodness of Fit Indices of the CLPMs*

| Model | df | χ^2^ | p-value (χ^2)^ | CFI | RMSEA | SRMR |
| --- | --- | --- | --- | --- | --- | --- |
| Emotion Suppression | 14 | 29.33 | .010 | .97 | .07 | .04 |
| Cognitive Reappraisal | 14 | 24.12 | .040 | .97 | .06 | .05 |
| Avoidant Attachment | 6 | 21.99 | <.001 | .96 | .12 | .04 |
| Anxious Attachment | 6 | 105.98 | <.001 | .56 | .19 | .06 |

*Note.* Robust p-value and fit indices are reported.

**Table A2**

*Indirect Effects*

| Indirect Effect | | | | Estimate | SE | p | 95% CI |
| --- | --- | --- | --- | --- | --- | --- | --- |
| Model 1: Emotion suppression | | | | | | | |
|  | *Mediation effects* | | | | | | |
|  |  | Treatment ® T2 ES ® T3 DS | | .003 | .003 | .374 | -0.002, 0.010 |
|  |  | Treatment ® T3 ES ® T4 DS | | .000 | .004 | .945 | -0.009, 0.007 |
|  | *Reverse mediation effects* | | | | | | |
|  |  |  | Treatment ® T2 DS ® T3 ES | .007 | .006 | .238 | -0.002, 0.023 |
|  |  |  | Treatment ® T3 DS ® T4 ES | .014 | .010 | .155 | -0.003, 0.035 |
| Model 2: Cognitive Reappraisal | | | | | | | |
|  | *Mediation effects* | | | | | | |
|  |  | Treatment ® T2 CR ® T3 DS | | .000 | .002 | .916 | -0.004, 0.003 |
|  |  | Treatment ® T3 CR ® T4 DS | | .000 | .003 | .911 | -0.007, 0.009 |
|  | *Reverse mediation effects* | | | | | | |
|  |  | Treatment ® T2 DS ® T3 CR | | -.010 | .007 | .185 | -0.025, 0.002 |
|  |  | Treatment ® T3 DS ® T4 CR | | .001 | .005 | .848 | -0.008, 0.010 |
| Model 3: Avoidant Attachment | | | | | | | |
|  | *Mediation Effects* | | | | | | |
|  |  | Treatment ® T3 AV ® T4 DS | | -.001 | .003 | .871 | -0.008, 0.007 |
|  | *Reverse Mediation Effects* | | | | | | |
|  |  | Treatment ® T3 DS ® T4 AV | | .007 | .006 | .239 | -0.002, 0.021 |
| Model 4: Anxious Attachment | | | | | | | |
|  | *Mediation Effects* | | | | | | |
|  |  | Treatment ® T3 AX ® T4 DS | | .000 | .003 | .896 | -0.006, 0.007 |
|  | *Reverse Mediation Effects* | | | | | | |
|  |  | Treatment ® T3 DS ® T4 AX | | .016 | .016 | .336 | -0.018, 0.045 |

*Note.* DS = depression; ES = emotion suppression; CR = cognitive reappraisal; AV = avoidant attachment; AX = anxious attachment. Bootstrapped confidence intervals with 500 bootstraps.

**Figure A1**

*CLPMs for the Mechanisms of Change and Depressive Symptoms with Treatment Added as a Predictor*

**a) Emotion Suppression**

*Note.* Significant paths are highlighted in bold and the significant p-values after the Benjamini-Hochberg correction for multiple testing are depicted.

* *p* < .05. ** *p* < .01. *** *p* < .001

**b) Cognitive Reappraisal**

*Note.* Significant paths are highlighted in bold and the significant p-values after the Benjamini-Hochberg correction for multiple testing are depicted.

* *p* < .05. ** *p* < .01. *** *p* < .001

**c) Avoidant Attachment**

*Note.* Significant paths are highlighted in bold and the significant p-values after the Benjamini-Hochberg correction for multiple testing are depicted. The added covariances based on the modification indices were included.

* *p* < .05. ** *p* < .01. *** *p* < .001

**d) Anxious Attachment**

*Note.* Significant paths are highlighted in bold and the significant p-values after the Benjamini-Hochberg correction for multiple testing are depicted. The added covariances based on the modification indices were included.

* *p* < .05. ** *p* < .01. *** *p* < .001

1. Note that in the analysis plan we specified that we would apply the Bonferroni-Holm correction for multiple testing. However, after further consideration, we found this correction would have been too conservative as the Benjamini-Hochberg correction is preferred when a larger number of hypotheses are tested simultaneously.
